# Ratiometric Molecular Probes Based on Dual Emission of a Blue Fluorescent Coumarin and a Red Phosphorescent Cationic Iridium(III) Complex for Intracellular Oxygen Sensing

**DOI:** 10.3390/s150613503

**Published:** 2015-06-09

**Authors:** Toshitada Yoshihara, Saori Murayama, Seiji Tobita

**Affiliations:** Department of Chemistry and Chemical Biology, Graduate School of Science and Technology, Gunma University, 1-5-1 Tenjin-cho, Kiryu, Gunma 376-8515, Japan; E-Mails: yoshihara@gunma-u.ac.jp (T.Y.); smurayama3580@yahoo.co.jp (S.M.)

**Keywords:** oxygen sensor, fluorescence, phosphorescence, iridium complex, ratiometric probe, living cells, imaging

## Abstract

Ratiometric molecular probes **RP1** and **RP2** consisting of a blue fluorescent coumarin and a red phosphorescent cationic iridium complex connected by a tetra- or octaproline linker, respectively, were designed and synthesized for sensing oxygen levels in living cells. These probes exhibited dual emission with good spectral separation in acetonitrile. The photorelaxation processes, including intramolecular energy transfer, were revealed by emission quantum yield and lifetime measurements. The ratios ($R_{\text{I}}=(I_{\text{p}}/I_{\text{f}})$) between the phosphorescence ($I_{\text{p}}$) and fluorescence ($I_{\text{f}}$) intensities showed excellent oxygen responses; the ratio of $R_{\text{I}}$ under degassed and aerated conditions ($R_{\text{I}}^{0}/R_{\text{I}})$ was 20.3 and 19.6 for **RP1** and **RP2**. The introduction of the cationic Ir (III) complex improved the cellular uptake efficiency compared to that of a neutral analogue with a tetraproline linker. The emission spectra of the ratiometric probes internalized into living HeLa or MCF-7 cells could be obtained using a conventional microplate reader. The complex **RP2** with an octaproline linker provided ratios comparable to the ratiometric measurements obtained using a microplate reader: the ratio of the $R_{\text{I}}$ value of **RP2** under hypoxia (2.5% O_2_) to that under normoxia (21% O_2_) was 1.5 and 1.7 for HeLa and MCF-7 cells, respectively. Thus, the intracellular oxygen levels of MCF-7 cells could be imaged by ratiometric emission measurements using the complex **RP2**.

## 1. Introduction

Molecular oxygen is critical in cell metabolism and is a key substrate in energy generation in aerobic organisms [1], serving as the terminal electron acceptor in the mitochondrial respiratory chain [2]. Oxygen deprivation has a large effect on numerous cellular enzymatic reactions involving cell signaling and genetic adaptation [3]. The detection of oxygen levels in living cells is therefore of great importance not only in cell biology, but also in physiology and pathophysiology.

Hypoxia imaging probes containing a *p*-nitrobenzyl [4,5], indolequinone [6], or azo [7,8] moiety have been developed to detect hypoxic cells and tissues. The fluorescence spectra or intensites of these probes are altered upon their enzymatic reduction under hypoxic conditions, and are thus useful for detecting hypoxic regions of cells and tissues. However, these probes are not suitable for real-time oxygen sensing of living cells and tissues because the enzymatic reactions under hypoxia are irreversible.

Oxygen probes based on the phosphorescence quenching of transition metal complexes by molecular oxygen can be used for real-time monitoring of intracellular or *in vivo* oxygen levels because oxygen quenching occurs reversibly in both cells and tissues [9,10,11,12,13,14,15,16,17]. Recently, many phosphorescent metal complexes, such as platinum(II) [18,19,20,21,22,23,24,25,26,27,28,29,30,31,32] or palladium(II) [33,34,35] porphyrins, and cyclometalated ruthenium(II) [36,37,38,39,40], platinum(II) [41,42,43] and iridium(III) [44,45,46,47,48,49,50,51,52,53,54,55] complexes have been developed for the measurement of cellular oxygen levels and/or the imaging of hypoxic tumors. The oxygen levels have usually been derived from the phosphorescence lifetime of the probes, which is a function of ambient oxygen concentration. However, although lifetime-based measurement is useful for quantifying oxygen levels, it requires specialized techniques, including a pulsed light source and a time-resolved detection system.

**Scheme 1:**
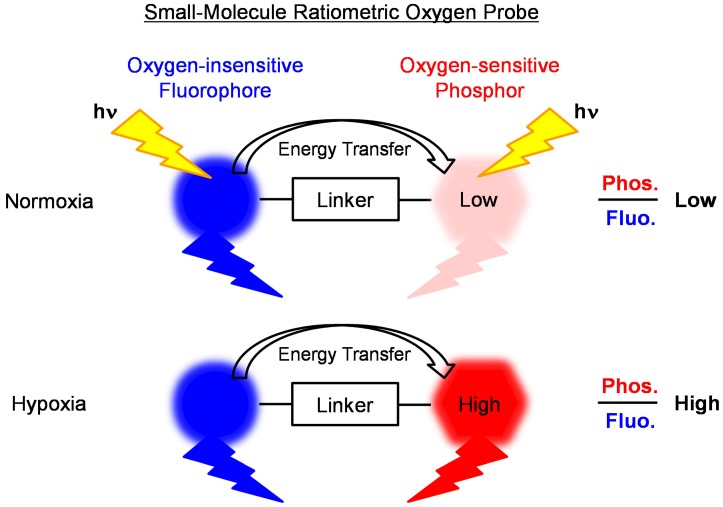
Design concept of a small-molecule ratiometric probe for sensing oxygen levels in living cells.

As an alternative, the ratiometric method does not require specialized equipment for measuring lifetimes and can be used for the real-time detection and imaging of oxygen levels in living cells and tissues. Ratiometric oxygen sensors based on small polymer particles or semiconductor nanocrystals containing an oxygen-insensitive fluorophore and oxygen-sensitive phosphorescent dye have been designed and synthesized for biological oxygen sensing [44,56,57,58,59,60,61,62,63,64,65,66,67,68,69,70]. These nanoparticles can be incorporated into living cells and demonstrate dual emission. However, these nanoparticles generally require longer incubation times to uptake into living cells compared with those of small molecules. Ratiometric oxygen sensors can also be based on small molecules [71,72]. Scheme 1 illustrates the design concept of a small-molecule ratiometric oxygen probe for sensing oxygen levels in living cells. The probe consists of an oxygen-insensitive fluorophore and oxygen-sensitive phosphor, which are connected by a rigid linker. These probes have the advantage of higher affinity to biological cells compared to nanoparticles. Moreover, these probe molecules can be chemically modified to improve not only their physicochemical properties, such as solubility, stability, and hydrophilicity, but also their photophysical properties, such as absorption and emission spectra, emission quantum yields, and lifetime.

Our ratiometric molecular oxygen probe (C343-Pro_4_-BTP) consisted of a blue fluorescent coumarin 343 (C343), and a red phosphorescent iridium(III) complex, BTP (bis(2-(2′-benzothienyl)-pyridinato-N,C^3’^)Ir(acetylacetonate)) [72]. The C343 and BTP moieties were connected by a rigid tetraproline linker. C343-Pro_4_-BTP showed dual emission due to C343 fluorescence and BTP phosphorescence, and the intensity ratio exhibited excellent oxygen response in both acetonitrile and lipid membranes. In addition, we noted that C343-Pro_4_-BTP holds promise as an optical sensor for visualizing the oxygen levels in living cells, although the oxygen concentration gradient in cultured cells could not be obtained due to its low cellular uptake efficiency.

**Figure 1:**
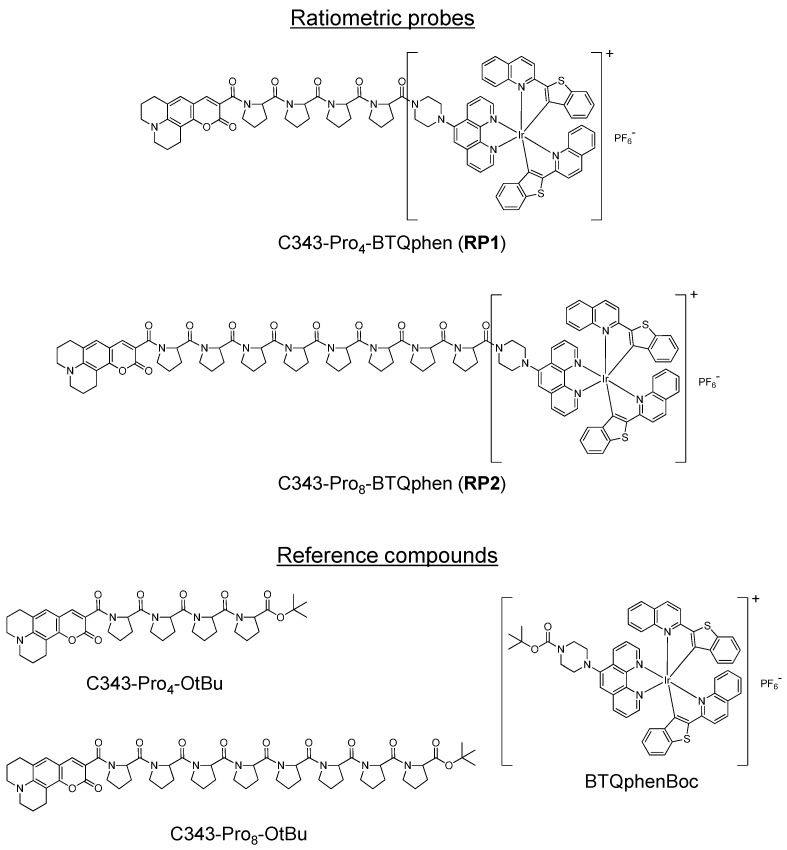
Chemical structures of the ratiometric probes C343-Pro_4_-BTQphen (**RP1**) and C343-Pro_8_-BTQphen (**RP2**) and their reference compounds, C343-Pro_4_-OtBu, C343-Pro_4_-OtBu, and BTQphenBoc.

In this study, we report the synthesis, photophysical properties, and oxygen-sensing properties of two new ratiometric oxygen probes, (C343-Pro_4_-BTQphen (**RP1**) and C343-Pro_8_-BTQphen (**RP2**)), bearing a cationic Ir(III) complex (BTQphen) in place of the neutral complex (BTP) in C343-Pro_4_-BTP (Figure 1). The Ir complex BTQphen was chosen as an oxygen-sensitive chromophore because cationic iridium complexes are usually more efficiently internalized into living cells compared to neutral Ir(III) complexes [45,46]; in addition, BTQphen phosphoresces at much longer wavelengths compared to the fluorescence band of C343. The new ratiometric oxygen sensors **RP1** and **RP2** exhibited improved cellular uptake efficiencies, and the ratiometric images of living cells responded clearly to the oxygen concentration of the cell incubator.

## 2. Experimental Section

### 2.1. Materials and Instruments

The reference compounds in Figure 1 were synthesized and identified as described in the Supplementary Information. Acetonitrile (MeCN, spectrophotometric grade, >99.7%, Kanto Chemical) was used as received. All reagents and solvents for the synthesis of **RP1** and **RP2** were purchased from Kanto Chemical (Tokyo, Japan), Wako Pure Chemical (Osaka, Japan), Tokyo Chemical Industries (Tokyo, Japan), or Sigma-Aldrich (Tokyo, Japan), and were used without further purification.

^1^H-NMR chemical shifts were recorded on a JNM-AL300 (JEOL) at 300 MHz or a JNM-ECS400 (JEOL, Tokyo, Japan) at 400 MHz using tetramethylsilane as an internal standard. ESI-MS and MALDI-TOF-MS measurements were carried out on API 2000 (Applied Biosystems Tokyo, Japan) and AXIMA Performance (Shimadzu, Kyoto, Japan) mass spectrophotometers. UV-Vis absorption spectra were recorded on a UV-Vis spectrophotometer (Ubest-550, JASCO, Tokyo, Japan). Emission spectra were measured with a photonic multichannel analyzer PMA-12 (C10027-01, Hamamatsu, Hamamatsu, Japan) equipped with a monochromatized Xe arc lamp. The emission spectrum was corrected for spectral sensitivity. Nitrogen-oxygen mixed gas regulated with a mass-flow meter (SEC-V110DM, PAC-D2, HORIBA STEC, Kyoto, Japan) was bubbled through the sample solution for 30 min prior to emission measurement, and then, the emission spectra were measured under different oxygen concentrations. Fluorescence and phosphorescence quantum yields were determined on an absolute photoluminescence quantum yield measurement system (C9920-01, Hamamatsu) consisting of a Xe arc lamp, a monochromater, an integrating sphere, and a multichannel detector [73]. Picosecond fluorescence lifetime measurements were carried out using a femtosecond laser system based on a mode-locked Ti:sapphire laser (Tsunami; center wavelength, 800 nm; pulse width, ca. 70 fs; repetition rate, 82 MHz, Spectra-Physics, Tokyo, Japan) pumped by a CW green laser (Millennia V; 532 nm, 4.5 W, Spectra-Physics) [74]. The generation of the second harmonic (400 nm, pulse width, ca. 200 fs) was performed in an LBO crystal. The third harmonic (266 nm, pulse width, ca. 250 fs) was generated by a sum frequency mixing of the fundamental and the second harmonic of the Tsunami laser system. The repetition frequency of the excitation pulse was reduced to 4 MHz using a pulse picker (Model 3980, Spectra-Physics). The second harmonic (400 nm) in the output beam was used as a trigger pulse. The emission from the sample solution was observed through a polarizer at the magic angle (54.7°) with respect to the polarization direction of the excitation laser pulse. The detection system consisted of a microchannel plate photomultiplier tube (R3809U-51, Hamamatsu) and a single-photon counting module (SPC-530, Becker and Hickl). The instrument response function had a half width of about 25 ps. The fluorescence decay curves were analyzed by deconvolution. Phosphorescence lifetimes were measured with a time-correlated single-photon counting (TCSPC) system (Quntaurus-Tau, Hamamatsu).

### 2.2. Syntheses of **RP1** and **RP2**

C343-Pro_4_-phen (46 mg, 0.05 mmol) or C343-Pro_8_-phen (65 mg, 0.05 mmol) and the chloro-bridged dimer of BTQ (38 mg, 0.025 mmol) were dissolved in dichloromethane (10 mL) and methanol (10 mL) and the solution was refluxed for 4 h (see Supplementary Information). After cooling, KPF_6_ (11 mg, 0.06 mmol) was added, the solution was stirred for 1 h, then the solution was dried under reduced pressure. The crude product was purified by aluminum column chromatography using chloroform:methanol (98:2 v/v) as eluent. Further purification was carried out using an HPLC system (Shimadzu) and the following conditions: column, YMC-Pack ODS-AM (300 × 6.0 mm); eluent, 50%–100% MeCN aq containing 0.1% TFA; flow rate, 1 mL/min. The main peak fraction was collected and purified water was added. The aqueous solution was dried by vacuum freezing to afford **RP1** or **RP2** as an orange powder (55 mg, 62% and 70 mg, 65%, respectively). MALDI-TOF (*m/z*) of **RP1**: calcd. for C_86_H_77_IrN_11_O_7_S_2_ [M-PF_6_^−^]^+^: 1632.51, found: 1632.21. MALDI-TOF (*m/z*) of **RP2**: calcd. for C_106_H_105_IrN_15_O_11_S_2_ [M-PF_6_^−^]^+^: 2020.72, found: 2020.51.

### 2.3. Cell Culture

Human uterine cancer-derived HeLa cells and human breast cancer-derived MCF-7 cells were purchased from the American Type Culture Collection and cultured in Dulbecco’s Modified Eagle Medium (DMEM) with 10% fetal bovine serum, penicillin (50 units/mL), and streptomycin (50 µg/mL). All cells were grown at 37 °C under a 5% CO_2_ atmosphere. An O_2_ concentration-changeable multigas incubator (Ex 9200, Wakenyaku, Kyoto, Japan or INUB-ONICS-F1-H2, GM-8000, TOKAI HIT, Fujinomiya, Japan) was used for 2.5% O_2_ incubation of cells.

### 2.4. Emission Imaging

The cells were seeded on glass-bottomed dishes, allowed to adhere for 24 h, and then were incubated with one of the ratiometric probes at the indicated concentrations for the indicated durations. An inverted microscope (IX71, Olympus), Tokyo, Japan equipped with a ×40 oil-immersion objective lens and an electron multiplying CCD camera (Evolve 512, PHOTOMETRICS, Tokyo, Japan) driven by MetaMorph software was used to obtain luminescence microscope images. A dual view simultaneous-imaging system (DV2, PHOTOMETRICS) was used for ratiometric imaging. Samples were excited using a 150 W mercury lamp.

### 2.5. Fluorescence and Phosphorescence Spectra in Living Cells

HeLa or MCF-7 cells (3.0 × 10^4^ cells/well) were seeded into a 96-well flat bottom plate and allowed to adhere for 5 h. The stock solutions of **RP1** and **RP2** were diluted with DMEM containing 10% fetal bovine serum, penicillin (50 units/mL), and streptomycin (50 µg/mL). The cells were incubated with the ratiometric probes for 12 h at 37 °C under a 5% CO_2_ atmosphere, then the medium was removed and the cells were washed twice with Hank’s balanced salt solution (HBSS, Gibco, Tokyo, Japan). The emission signals of each probe were measured using a microplate reader (Infinite 200 Pro, Tecan, Kawasaki, Japan) equipped with a gas controlled module (GCM, Tecan).

## 3. Results and Discussion

### 3.1. Photophysical Properties in Solution

**Figure 2:**
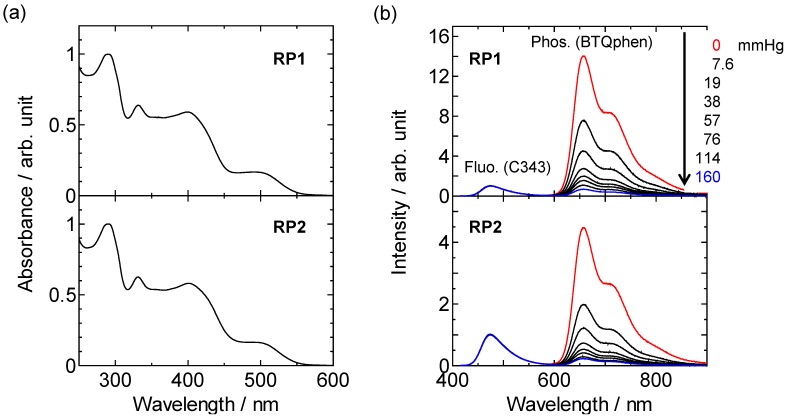
(**a**) Absorption spectra of **RP1** and **RP2** in MeCN. (**b**) Emission spectra of **RP1** and **RP2** in MeCN under different oxygen partial pressures (*λ*_ex_ = 405 nm).

We first examined the photophysical properties of **RP1** and **RP2** together with the reference compounds that possess only the C343 or BTQphen unit (Figure 1) to clarify the relaxation processes of **RP1** and **RP2** in the excited state. The absorption spectra of **RP1** and **RP2** in MeCN displayed very similar spectral shapes and positions, as shown in Figure 2a. The first absorption band of **RP1** and **RP2**, with the maximum at around 500 nm, can be assigned to the electronic transitions of the first singlet metal-to-ligand charge transfer state of the BTQphen moiety. The second absorption band, with the maximum at around 400 nm, is composed of the ππ* absorption bands of the C343 and BTQphen chromophores, judging from the absorption spectra of the reference compounds, C343-Pro_4_-OtBu, C343-Pro_8_-OtBu, and BTQphenBoc in MeCN (Figure S1 in Supplementary Information). It can be seen from Figure S1 that the C343 and BTQphen moieties are simultaneously excited at 405 nm, the excitation wavelength used in this study.

Figure 2b shows the emission spectra of **RP1** and **RP2** in degassed and aerated MeCN following excitation at 405 nm. Both compounds exhibit two emission bands originating from C343 fluorescence with a maximum at 477 nm and BTQphen phosphorescence with a maximum at 657 nm. Spectral separation is fairly good, which is advantageous for ratiometric measurements. The phosphorescence intensity is much higher under the degassed condition (red lines) than the fluorescence intensity in both compounds, giving a greater intensity ratio in the complex **RP1**. The fluorescence intensities of **RP1** and **RP2** under the aerated condition (blue lines) are similar to those under the degassed condition (red lines), whereas the phosphorescence intensities dramatically decrease under the aerated condition, thus demonstrating the good oxygen responses of **RP1** and **RP2** in MeCN.

To clarify the relaxation processes of electronically excited **RP1** and **RP2**, we measured the emission quantum yields and lifetimes of the complexes **RP1** and **RP2** and their reference compounds. The photophysical parameters obtained for these compounds are summarized in Table 1. The fluorescence quantum yields (${\text{Φ}}_{\text{f}}^{0}$) of C343-Pro_4_-OtBu and C343-Pro_8_-OtBu in degassed MeCN were extremely large (0.94 and 0.93, respectively), whereas those of **RP1** and **RP2** were reduced to 0.009 and 0.023, suggesting that efficient energy transfer from C343 to the BTQphen unit is involved in the relaxation processes of excited **RP1** and **RP2**. Intermolecular energy transfer due to collisions in the excited singlet state is unlikely to occur under the present solute concentrations (5 µM). It is also unlikely that electron transfer contributes to the quenching of C343 fluorescence by BTQphen because the phosphorescence quantum yields of **RP1** and **RP2** under the degassed condition are comparable to that of BTQphenBoc. The good spectral overlap of the fluorescence spectrum of C343 and the absorption spectrum of BTQphen (see Figure 2) suggests that Förster resonance energy transfer is the most probable energy transfer mechanism for **RP1** and **RP2**.

**Table 1:** Photophysical parameters of **RP1**, **RP2**, and reference compounds in MeCN (λ_ex_ = 405 nm).

Compound	${\text{Φ}}_{\text{f}}^{0}$	${\text{Φ}}_{\text{p}}$	${\text{Φ}}_{\text{p}}^{0}$	${\text{τ}}_{\text{f}}^{0}$/ns ^(a)^	${\text{τ}}_{\text{p}}$/µs ^(b)^	${\text{τ}}_{\text{p}}^{0}$/µs ^(b)^	${\text{Φ}}_{\text{SET}}$
**RP1**	0.009	0.016	0.29	0.057 (59%) ^(c)^ 0.14 (41%) ^(c)^	0.28	5.9	0.97
**RP2**	0.023	0.016	0.28	0.16 (59%) ^(c)^ 0.61 (41%) ^(c)^	0.28	5.8	0.89
C343-Pro_4_-OtBu	0.94	-	-	4.0	-	-	
C343-Pro_8_-OtBu	0.93	-	-	4.0	-	-	
BTQphenBoc	-	0.011	0.20	-	0.29	5.4	

^(a)^ monitored at 480 nm; ^(b)^ monitored at 660 nm; ^(c)^ relative intensities calculated by pre-exponential factors.

**Figure 3:**
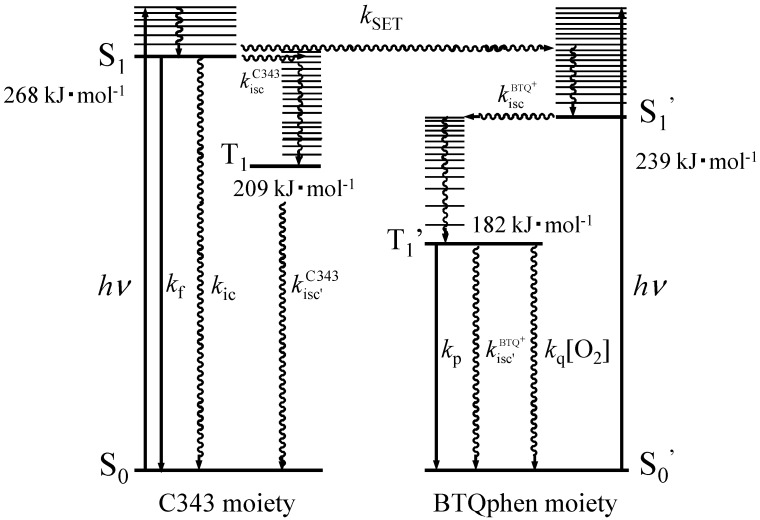
Energy state diagram and relaxation processes of excited singlet (S_1_) and triplet (T_1_) states of C343-Pro_4_-BTQphen and C343-Pro_8_-BTQphen. *k*_isc_, *k*_isc_^,^, *k*_f_, *k*_ic_, *k*_SET_, *k*_p_ and *k*_q_ are the rate constants for S_1_→T_1_ and T_1_→S_0_ intersystem crossing, fluorescence, internal conversion, singlet energy transfer, phosphorescence, and quenching, respectively.

Given the involvement of intramolecular energy transfer, we next measured the fluorescence lifetimes of **RP1** and **RP2** to evaluate their energy transfer efficiencies. The fluorescence decay curves of **RP1** and **RP2** monitored at 480 nm could be fitted to biexponential decay functions with lifetimes of τ_f1_ = 57 ps (*A*_1_ = 0.59) and τ_f2_ = 140 ps (*A*_2_ = 0.41), and τ_f1_ = 160 ps (*A*_1_ = 0.59) and τ_f2_ = 610 ps (*A*_2_ = 0.41), respectively, where *A*_1_ and *A*_2_ denote the pre-exponential factors. These lifetimes are much shorter than that (4.0 ns) of the reference compounds C343-Pro_4_-OtBu and C343-Pro_8_-OtBu. From the amplitude-averaged lifetime <τ_f_>, calculated using <τ_f_> = *A*_1_τ_f1_ + *A*_2_τ_f2_ for **RP1** and **RP2**, the efficiencies of the Förster resonance energy transfer in the excited singlet state (${\text{Φ}}_{\text{SET}}$) were estimated to be 97% for **RP1** and 89% for **RP2**. This difference in the ${\text{Φ}}_{\text{SET}}$ values can be attributed to the difference in the number of proline residues in **RP1** and **RP2**, *i.e.*, the distance between the C343 and BTQphen luminophores. The observation of two distinct lifetime components for **RP1** and **RP2** suggests the presence of at least two different conformers in MeCN. As a possible explanation, it can be invoked that oligoprolines exist in two helical conformations [75,76,77,78]: one is a type I *cis* helix conformer generated with the shorter chain, and the other is a type II *trans* helix conformer generated with the longer chain. Thus, it is expected that the shorter-lifetime component corresponds to the type I *cis* helix and the longer-lifetime component corresponds to the type II *trans* helix. In contrast to the fluorescence lifetime, the phosphorescence lifetimes of **RP1** and **RP2** under the degassed condition (${\text{τ}}_{\text{p}}^{0}$) were almost equal to that of the reference compound BTQphenBoc, showing that phosphorescence quenching due to reverse energy transfer and/or electron transfer is negligible in the excited triplet state. From the absorption and emission spectra and the photophysical data of **RP1** and **RP2**, the photorelaxation processes of **RP1** and **RP2** can be represented in terms of an energy state diagram as shown in Figure 3. It can be understood from Figure 3 that reverse energy transfer from BTQphen to C343 does not occur in the excited triplet state, which is beneficial for ratiometric molecular probes.

### 3.2. Oxygen Responses in Solution

The phosphorescence quantum yields of **RP1** and **RP2** under the degassed and aerated conditions (${\text{Φ}}_{\text{p}}^{0}$ and ${\text{Φ}}_{\text{p}}$) obtained following 405 nm excitation were 0.29 and 0.016, and 0.28 and 0.016, respectively, indicating that the phosphorescence of BTQphen is efficiently quenched by dissolved oxygen in MeCN. The oxygen responses of **RP1** and **RP2** in MeCN, which were evaluated by the ratio (${\text{τ}}_{\text{p}}^{0}/{\text{τ}}_{\text{p}}$) of the phosphorescence lifetimes taken under the degassed and aerated conditions, were 21.1 and 20.7, respectively. These values are close to those (18.1 for **RP1**, 17.5 for **RP2**) evaluated from the ratio (${\text{Φ}}_{\text{p}}^{0}/{\text{Φ}}_{\text{p}}$) of the phosphorescence quantum yield, although the oxygen responses evaluated by (${\text{Φ}}_{\text{p}}^{0}/{\text{Φ}}_{\text{p}}$) are slightly lower than those evaluated by (${\text{τ}}_{\text{p}}^{0}/{\text{τ}}_{\text{p}}$). This difference is attributable to the energy transfer efficiency from C343 to BTQphen being less than unity.

In ratiometric measurements we use the ratio ($R_{\text{I}}\;=I_{\text{p}}/I_{\text{f}}$) between the phosphorescence intensity ($I_{\text{p}}$) at 657 nm and the fluorescence intensity ($I_{\text{f}}$) at 477 nm under given oxygen conditions and the ratio ($R_{\text{I}}^{0}=\;I_{\text{p}}^{0}/I_{\text{f}}^{0}$) under the degassed condition. The $R_{\text{I}}^{0}$ values of **RP1** and **RP2** were determined to be 14 and 4.5, and the $R_{\text{I}}$ values under the aerated condition were ascertained to be 0.69 and 0.23, respectively. The $R_{\text{I}}^{0}$ value of **RP2** is much smaller than that of **RP1**, suggesting that the energy transfer rate from the excited C343 to the BTQphen chromophore in **RP2** is slower than that in **RP1**. This was confirmed by the lifetime measurements described above and is consistent with the longer distance between the fluorophore and the phosphor in **RP2**. It should be noted that the $R_{\text{I}}^{0}/R_{\text{I}}$ values of **RP1** (20.3) and **RP2** (19.6) in MeCN are almost the same because the oxygen response is governed by the oxygen quenching properties of the BTQphen phosphor.

### 3.3. Cellular Uptake Efficiencies

**Figure 4:**
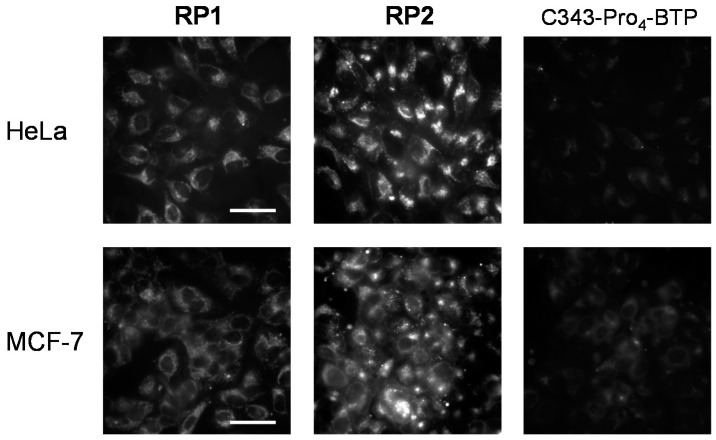
Emission images of HeLa and MCF-7 cells incubated with **RP1**, **RP2**, or C343-Pro_4_-BTP (2 µM) for 4 h at 37 °C. Scale bar: 50 µm.

We then examined the cellular uptake properties of **RP1** and **RP2**, and that of C343-Pro_4_-BTP, which we previously synthesized [72]. Each probe was added to the medium of HeLa or MCF-7 cells at a final concentration of 2 µM, the cells were incubated for 4 h, then washed with medium without phenol red. The excitation wavelength was 400–410 nm. Figure 4 displays the emission images of the living cells observed in the wavelength range over 455 nm, *i.e.*, the total emission of the probes. The emission images of **RP1** and **RP2** in both cell lines are much brighter than those of C343-Pro_4_-BTP, suggesting that the cellular uptake efficiencies of **RP1** and **RP2** are significantly enhanced by replacing the neutral BTP in C343-Pro_4_-BTP with a cationic BTQphen. This is consistent with our observation that the cellular uptake efficiencies of cationic iridium complexes with a 5-dialkylamino-1,10-phenanthroline ligand are much larger than those of the neutral analogues with an acetylacetonato ligand [46]. Very recently, Tanabe and co-workers have synthesized a water-soluble ratiometric oxygen probe comprising a fluorescent coumarin and a phosphorescent ruthenium complex [71]. The cellular uptake efficiency of this probe seems to be lower than those of our probes, because they have added a much higher concentration of probe (100 µM) into the culture medium compared with that (2 µM) of our probes.

### 3.4. Oxygen Responses in Living Cells

**Figure 5:**
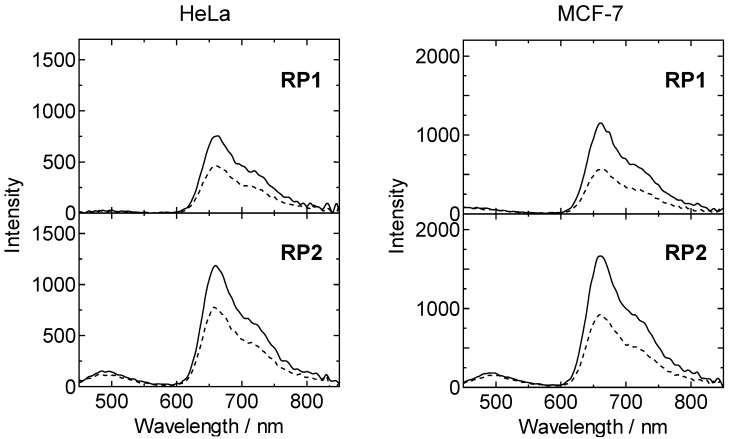
Emission spectra of **RP1** and **RP2** internalized into HeLa or MCF-7 cells under 21% (dashed line) and 2.5% (solid line) O_2_ conditions. Concentration, 5 µM; incubation time, 12 h; λ_ex_, 405 nm.

We next investigated the oxygen responses of **RP1** and **RP2** in living cells by using a microplate reader equipped with a gas control module. Figure 5 shows the emission spectra of **RP1** and **RP2** incorporated into HeLa or MCF-7 cells under 21% and 2.5% O_2_ conditions. In both cell lines, the complex **RP1** gives an essentially single emission band with a maximum at 660 nm. This emission can be ascribed to the phosphorescence of BTQphen. In contrast with **RP1**, the complex **RP2** gives a dual emission band with maxima at 480 nm and 660 nm, originating from the fluorescence of C343 and the phosphorescence of BTQphen. To the best of our knowledge, there have been very few reports of dual emission spectra of ratiometric probes in living cells obtained using a microplate reader.

The $R_{\text{I}}$ values of **RP2** in HeLa cells under 21% and 2.5% O_2_ conditions were determined to be 5.9 and 9.1, and those in MCF-7 cells to be 7.0 and 11.7, respectively. The ratios of the $R_{\text{I}}$ value of **RP2** under hypoxia (2.5% O_2_) to that under normoxia (21% O_2_) ($R_{\text{I}}\left(\text{2.5\%}\right)/R_{\text{I}}\left(\text{21\%}\right))$ were 1.5 and 1.7 for HeLa and MCF-7 cells, respectively. To confirm the reliability of the oxygen responses of **RP2** in living cells we measured the phosphorescence lifetime of **RP2** in HeLa and MCF-7 cells under 21% and 2.5% O_2_ conditions by using a time-correlated single-photon counting system [45] (Figure S2 and Table S1 in Supplementary Information). The intensity-averaged lifetimes <τ_p_>, calculated using <τ_p_> = (*A*_1_τ_p1_^2^ + *A*_2_τ_p2_^2^)/(*A*_1_τ_p1_ + *A*_2_τ_p2_) for **RP2** in HeLa cells under 21% and 2.5% O2 conditions, were obtained to be 2.80 µs and 4.05 µs, and those in MCF-7 cells to be 2.62 µs and 4.34 µs, respectively. The ratios (<τ_p_(2.5%)>/<τ_p_(21%)>) of the phosphorescence lifetimes were determined to be 1.45 for HeLa cells and 1.66 for MCF-7 cells, respectively. These values are in good agreement with $R_{\text{I}}\left(\text{2.5\%}\right)/R_{\text{I}}\left(\text{21\%}\right)$ obtained by the ratiometric measurements using **RP2**, demonstrating the reliability of ratiometric oxygen measurements using **RP2** as an oxygen sensor for living cells. Furthermore, to evaluate the oxygen response of **RP2** in living cells, we compared the oxygen responses of **RP2** and a commercially available oxygen probe (MitoXpress^®^-Intra NanO2) [24] by measuring the phosphorescence lifetimes of MitoXpress^®^-Intra in HeLa cells using the time-resolved fluorescence (TR-F) mode of the microplate reader. MitoXpress^®^-Intra is a phosphorescent nanoparticle containing Pt(II)-porphyrin. The ${\text{τ}}_{\text{p}}$ values of MitoXpress^®^-Intra in HeLa cells under 21% and 2.5% O_2_ conditions were determined to be 34 µs and 53 µs, respectively, giving a ratio (${\text{τ}}_{\text{p}}\left(\text{2.5\%}\right)/{\text{τ}}_{\text{p}}\left(\text{21\%}\right)$) of the phosphorescence lifetime of 1.6. This value is very close to that obtained by the ratiometric measurements using **RP2**, indicating that the oxygen response of **RP2** in living cells is comparable to that of MitoXpress^®^-Intra.

### 3.5. Ratiometric Imaging of Oxygen Levels in Living Cells

**Figure 6:**
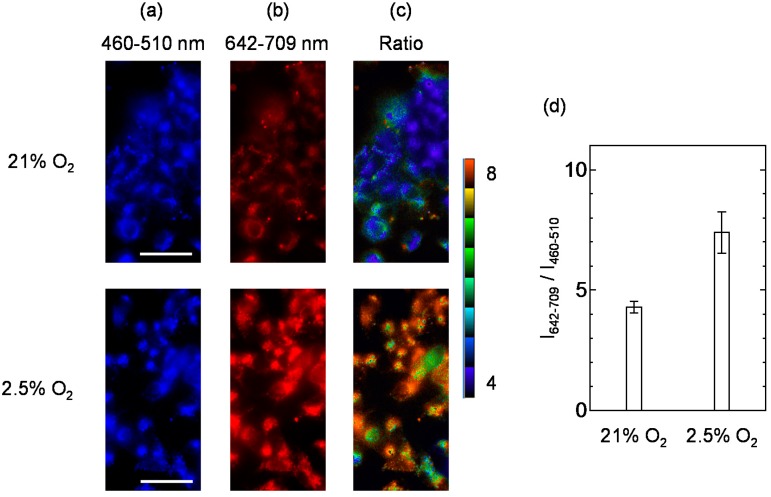
Emission images of MCF-7 cells treated with **RP2** (2 µM) for 12 h at 37 °C and incubated at different oxygen conditions (21% and 2.5% O_2_). The signals were received in two channels ((**a**) 460–510 nm and (**b**) 642–709 nm) following excitation at 400–410 nm. (**c**) Ratiometic images obtained from the phosphorescence intensity at 642–709 nm to that of the fluorescence intensity at 460–510 nm under 21% and 2.5% O_2_ conditions. Scale bar, 50 µm. (**d**) $I_{\text{642-709}}/I_{\text{460-510}}$ of **2** in MCF-7 cells (n = 6) under 21% and 2.5% O_2_ conditions.

Ratiometric imaging experiments were conducted to examine intracellular oxygen levels in MCF-7 cells. MCF-7 cells were cultured under the 21% O_2_ condition for 24 h at 37 °C. Ratiometric probe **RP2** was added to the medium at a final concentration of 2 µM, the cells were incubated for 12 h, then washed with HBSS. First, images of the MCF-7 cells were obtained under the 21% O_2_ condition, then the MCF-7 cells were incubated for 2 h under the 2.5% O_2_ condition. The excitation wavelength was 400–410 nm. In our previous report on C343-Pro_4_-BTP [72], we first obtained the fluorescence image and then the phosphorescence image by using a different filter cube. Here, we simultaneously obtained the fluorescence and phosphorescence images by using a dual view simultaneous-imaging system. Figure 6a shows the emission images of MCF-7 cells observed in the wavelength range from 460 nm to 510 nm, which corresponds to the fluorescence of C343. The fluorescence intensities from the MCF-7 cells are almost the same under 21% and 2.5% O_2_ conditions. In contrast, as shown in Figure 6b, the emission images taken at wavelength range from 642 nm to 709 nm, which correspond to the phosphorescence of BTQphen, show a brighter image under the 2.5% O_2_ condition than under the 21% O_2_ condition. The ratiometric images (Figure 6c) are predominantly blue-green in color under the 21% O_2_ condition, indicating low ratio values, whereas predominantly light-green to orange color is observed under the 2.5% O_2_ condition, representing high ratio values.

We calculated the ratio ($R_{\text{I}}\;=\;\left(I_{\text{642-709}}/I_{\text{460-510}}\right)$) between the fluorescence and phosphorescence intensities of **RP2** in MCF-7 cells (n = 6) under 21% and 2.5% O_2_ conditions (Figure 6d). The average ratio of the $R_{\text{I}}$ value of **RP2** under hypoxia (2.5% O_2_) to that under normoxia (21% O_2_) was determined to be 1.7, in overall agreement with the value obtained using a microplate reader. These results demonstrate that the complex **RP2** can be used as a ratiometric probe for oxygen sensing in living cells.

## 4. Conclusions

We designed and synthesized the new ratiometric oxygen sensors **RP1** and **RP2**, which consist of a blue fluorescent coumarin 343, and a red phosphorescent cationic iridium complex, BTPphen. A tetraproline or an octaproline linker was used to rigidly connect the fluorophore and phosphor in **RP1** and **RP2**, respectively. Spectral and photophysical measurements of **RP1** and **RP2** and their reference compounds showed that 405 nm excitation causes intramolecular energy transfer from C343 to BTPphen in the excited singlet states of **RP1** and **RP2**, resulting in dual emission originating from C343 fluorescence and BTPphen phosphorescence. The ratio ($R_{\text{I}}\;=I_{\text{p}}/I_{\text{f}}$) between the phosphorescence and fluorescence intensities of **RP1** and **RP2** was obtained in degassed ($R_{\text{I}}^{0}$) and aerated ($R_{\text{I}}$) MeCN and their ratios $R_{\text{I}}^{0}/R_{\text{I}}$ were determined to be 20.3 and 19.6, respectively, showing good oxygen sensitivity. The new ratiometric probes **RP1** and **RP2** showed higher cellular uptake efficiencies compared to their respective analogue bearing the neutral iridium complex BTP instead of the cationic BTPphen. The complex **RP2** internalized into HeLa or MCF-7 cells gave ratios of the $R_{\text{I}}$ value under hypoxia (2.5% O_2_) to that under normoxia (21% O_2_) comparable to the ratiometric measurements obtained using a microplate reader. In addition, the intracellular oxygen levels of MCF-7 cells could be imaged by ratiometric emission measurements using the complex **RP2**.

## Supplementary Files

Supplementary File 1
