# Supplementary material for: Ratiometric Molecular Probes Based on Dual Emission of a Blue Fluorescent Coumarin and a Red Phosphorescent Cationic Iridium(III) Complex for Intracellular Oxygen Sensing
Source: Sensors (Basel). 2015 Jun 9;15(6):13503–21. doi: 10.3390/s150613503 (PMC4507661; doi:10.3390/s150613503)
Supplement: Supplementary File 1 [file sensors-15-13503-s001.pdf]

OPEN ACCESS

sensors

ISSN 1424-8220
www.mdpi.com/journal/sensors

Supplementary Information

Ratiometric Molecular Probes Based on Dual Emission of a Blue Fluorescent Coumarin and a Red Phosphorescent Cationic Iridium(III) Complex for Intracellular Oxygen Sensing. *Sensors* 2015, *15*, 13503-13521

Toshitada Yoshihara, Saori Murayama and Seiji Tobita*

Department of Chemistry and Chemical Biology, Graduate School of Science and Technology, Gunma University, 1-5-1 Tenjin-cho, Kiryu, Gunma 376-8515, Japan;
E-Mail: yoshihara@gunma-u.ac.jp (T.Y.); smurayama3580@yahoo.co.jp (S.M.)

* Author to whom correspondence should be addressed; E-Mail: tobita@gunma-u.ac.jp;
Tel.: +81-277-30-1210; Fax: +81-277-30-1213.

1. Materials

Z-Pro$_2$-OtBu

N-carbobenzoxy-L-proline (Z-Pro-OH, 13.2 g, 53.0 mmol) and L-proline *tert*-butyl ester hydrochloride (10.0 g, 48.1 mmol) were dissolved in anhydrous dichloromethane, then 1-ethyl-3-(3-dimethylaminopropyl)-carbodiimide hydrochloride (EDC·HCl, 10.3 g, 53.7 mmol) and *N*,*N*-diisopropylethylamine (DIPEA, 9 mL, 52.9 mmol) were added. This solution was stirred for 1 h at 0 °C and then 20 h at room temperature. The reaction mixture was concentrated in vacuo and ethyl acetate was added. The organic layer was washed with 10% citric acid aqueous solution, saturated sodium hydrogen carbonate, and water. The organic layer was dried over sodium sulfate and was evaporated to dryness under reduced pressure. The product (Z-Pro$_2$-OtBu) was obtained as white powder (16.4 g, 41.3 mmol, 86%).^{1}H NMR (300 MHz, $CDCl_3$) δ: 7.35–7.28 (m, 5H), 5.20–4.97 (m, 2H), 4.55–4.27 (m, 2H), 3.80–3.31 (m, 4H), 2.21-1.88 (m, 8H), 1.44 (s, 9H).

H-Pro$_2$-OtBu

Z-Pro$_2$-OtBu (16.4 g, 41.3 mmol) was dissolved in methanol, and Pd/C was added slowly. The reaction vessel was filled with hydrogen gas. This solution was stirred for 6 h at room temperature and then evaporated to dryness under reduced pressure. The product (H-Pro$_2$-OtBu) was obtained as white powder (11.2 g, 41.0 mmol, 99%). ^{1}H NMR (300 MHz, $CDCl_3$) δ: 4.44-4.40 (m, 1H), 3.81–3.76 (m, 1H), 3.60–3.52 (m, 3H), 3.22–3.13 (m, 1H), 2.84–2.78 (m, 1H), 2.21–1.92 (m, 8H), 1.46 (s, 9H).

Z-Pro$_3$-OtBu

Z-Pro-OH (11.2 g, 44.9 mmol) and H-Pro$_2$-OtBu (11.2 g, 41.0 mmol) were dissolved in anhydrous dichloromethane, then EDC·HCl (8.7 g, 45.4 mmol) was added. This solution was stirred for 1 h at 0 °C and then 20 h at room temperature. The reaction mixture was concentrated in vacuo and ethyl acetate was added. The organic layer was washed with 10% citric acid aqueous solution, saturated sodium hydrogen carbonate, and water. The organic layer was dried over sodium sulfate and was evaporated to dryness under reduced pressure. The product (Z-Pro$_3$-OtBu) was obtained as white powder (16.8 g, 33.6 mmol, 82%).^{1}H NMR (300 MHz, $CDCl_3$) δ: 7.35–7.31 (m, 5H), 5.20–4.97 (m, 2H), 4.77–4.44 (m, 3H), 3.78–3.39 (m, 6H), 2.16-1.84 (m, 12H), 1.43 (s, 9H).

H-Pro$_3$-OtBu

Z-Pro$_3$-OtBu (16.8 g, 33.6 mmol) was dissolved in methanol, and Pd/C was added slowly. The reaction vessel was filled with hydrogen gas. This solution was stirred for 6 h at room temperature and then evaporated to dryness under reduced pressure. The product (H-Pro$_3$-OtBu) was obtained as white powder (12.0 g, 32.8 mmol, 98%). ^{1}H NMR (300 MHz, $CDCl_3$) δ: 4.73–4.69 (m, 1H), 4.50–4.46 (m, 1H), 3.82–3.76 (m, 2H), 3.66-3.56 (m, 4H), 3.18–3.10 (m, 1H), 2.83–2.75 (m, 1H), 2.22–1.93 (m, 12H), 1.44 (s, 9H).

Z-Pro$_4$-OtBu

Z-Pro-OH (9.4 g, 37.6 mmol) and H-Pro$_3$-OtBu (12.0 g, 32.8 mmol) were dissolved in anhydrous dichloromethane, then EDC·HCl (7.3 g, 38.0 mmol) was added. This solution was stirred for 1 h at 0 °C and then 20 h at room temperature. The reaction mixture was concentrated in vacuo and ethyl acetate was added. The organic layer was washed with 10% citric acid aqueous solution, saturated sodium hydrogen carbonate, and water. The organic layer was dried over sodium sulfate and was evaporated to dryness under reduced pressure. The product (Z-Pro$_4$-OtBu) was obtained as white powder (16.0 g, 26.8 mmol, 80%).^{1}H NMR (300 MHz, $CDCl_3$) δ: 7.36–7.30 (m, 5H), 5.22–4.98 (m, 2H), 4.78–4.43 (m, 4H), 3.76–3.37 (m, 8H), 2.16–1.83 (m, 16H), 1.43 (s, 9H).

H-Pro$_4$-OtBu

Z-Pro$_4$-OtBu (1.97 g, 3.3 mmol) was dissolved in methanol, and Pd/C was added slowly. The reaction vessel was filled with hydrogen gas. This solution was stirred for 6 h at room temperature and then evaporated to dryness under reduced pressure. The product (H-Pro$_4$-OtBu) was obtained as white powder (1.48 g, 3.2 mmol, 97%). ^{1}H NMR (400 MHz, $CDCl_3$) δ: 4.77–4.67 (m, 2H), 4.44–4.41 (m, 1H), 3.80–3.50 (m, 8H), 3.16–3.10 (m, 1H), 2.81–2.75 (m, 1H), 2.22–1.70 (m, 16H), 1.41 (s, 9H).

Z-Pro4-OH

Z-Pro4-OtBu (1.97 g, 3.3 mmol) was dissolved in 4M HCl/dioxane solution (15 mL). This solution was kept for 15 h at room temperature and then evaporated to dryness under reduced pressure. The product (Z-Pro4-OH) was obtained as white powder (1.73 g, 3.2 mmol, 97%). ^{1}H NMR (400 MHz, $CDCl_3$) δ: 7.40–7.27 (m, 5H), 5.19–4.96 (m, 2H), 4.76–4.41 (m, 4H), 3.84–3.45 (m, 8H), 2.31–1.80 (m, 16H).

Z-Pro8-OtBu

Z-Pro4-OtBu (1.62 g, 3.0 mmol) and H-Pro4-OtBu (1.39 g, 3.0 mmol) were dissolved in anhydrous dichloromethane, then EDC·HCl (690 mg, 3.6 mmol) and 1-hydroxybenzotriazole (HOBt, 505 mg, 3.3 mmol) were added. This solution was stirred for 1 h at 0 °C and then 20 h at room temperature. The reaction mixture was concentrated in vacuo and ethyl acetate was added. The organic layer was washed with 10% citric acid aqueous solution, saturated sodium hydrogen carbonate, and water. The organic layer was dried over sodium sulfate and was evaporated to dryness under reduced pressure. The product (Z-Pro4-OtBu) was obtained as white powder (2.38 g, 2.4 mmol, 80%). ^{1}H NMR (300 MHz, $CDCl_3$) δ: 7.37–7.27 (m, 5H), 5.19–4.96 (m, 2H), 4.78–4.71 (m, 6H), 4.58–4.40 (m, 2H), 3.78–3.36 (m, 16H), 2.13-1.81 (m, 32H), 1.41 (s, 9H). ESI-MS (Positive): calcd. for $C_{52}H_{72}N_8O_{11}$: 984.53, found: 1007.1 ($[M + Na]^+$).

H-Pro8-OtBu

Z-Pro8-OtBu (2.38 g, 2.4 mmol) was dissolved in methanol, and Pd/C was added slowly. The reaction vessel was filled with hydrogen gas. This solution was stirred for 6 h at room temperature and then evaporated to dryness under reduced pressure. The product (H-Pro8-OtBu) was obtained as white powder (2.04 g, 2.4 mmol, 100%). ^{1}H NMR (400 MHz, $CDCl_3$) δ: 4.76-4.66 (m, 6H), 4.43–4.40 (m, 1H), 3.92–3.49 (m, 16H), 3.18–3.12 (m, 1H), 2.90-2.84 (m, 1H), 2.19–1.70 (m, 32H), 1.41 (s, 9H).

C343-Pro4-OtBu (reference compound)

Coumarin343 (285 mg, 1.0 mmol), H-Pro4-OtBu (510 mg, 1.1 mmol), and 2-(1H-7-azabenzotriazol-1-yl)-1,1,3,3-tetramethyluronium hexafluorophosphate (HATU, 494 mg, 1.3 mmol) were dissolved in anhydrous *N,N'*-dimethylformamide (DMF, 30 mL), then DIPEA (1.0 mL) was added. This solution was stirred for 1 h at 0 °C and then 20 h at room temperature. The distilled water was added into the reaction mixture, and then the product was extracted by chloroform. This solution was dried over sodium sulfate and was evaporated to dryness under reduced pressure. The crude product was purified by silica-gel column chromatography using chloroform:methanol (19:1 v/v) as eluent. The product (C343-Pro4-OtBu) was obtained as yellow powder (438 mg, 0.60 mmol, 60%). ^{1}H NMR (400 MHz, $CDCl_3$) δ: 7.79 (s, 1H), 6.82 (s, 1H), 4.80–4.71 (m, 3H), 4.44–4.43 (m, 1H), 3.92–3.50 (m, 8H), 3.30–3.25 (m, 4H), 2.94–2.71 (m, 4H), 2.25–1.65 (m, 20H), 1.42 (s, 9H).

C343-Pro4-OH

C343-Pro4-OtBu (365 mg, 0.50 mmol) was dissolved in 4M HCl/dioxane solution (10 mL). This solution was kept for 15 h at room temperature and then evaporated to dryness under reduced pressure. The product (C343-Pro4-OH) was obtained as yellow powder (330 mg, 0.49 mmol, 98%). ^{1}H NMR (400 MHz, DMSO-*d6*) δ: 7.09 (s, 1H), 6.31 (s, 1H), 4.93–4.52 (m, 4H), 3.87–3.42 (m, 8H), 3.34–3.25 (m, 4H), 2.71–2.64 (m, 4H), 2.38–1.68 (m, 20H).

C343-Pro4-phen

C343-Pro4-OH (202 mg, 0.30 mmol), 5-(piperazin-1-yl)-1,10-phenanthroline hydrochloride (phen, 150 mg, 0.45 mmol), HOBt (75 mg, 0.49 mmol), and HATU (200 mg, 0.52 mmol) were dissolved in anhydrous DMF (10 mL), then DIPEA (0.2 mL) was added. This solution was stirred for 1 h at 0 °C and then 20 h at room temperature. The distilled water was added into the reaction mixture, and then the product was extracted by chloroform. This solution was dried over sodium sulfate and was evaporated to dryness under reduced pressure. The crude product was purified by aluminum column chromatography using chloroform:methanol (98:2 v/v) as eluent. The product (C343-Pro4-phen) was obtained as yellow powder (98 mg, 0.11 mmol, 37%). ^{1}H NMR (400 MHz, $CDCl_3$) δ: 9.19–9.18 (d, 1H), 9.08–9.01 (d, 1H), 8.57–8.54 (d, 1H), 8.13–8.11 (d, 1H), 7.79 (s, 1H), 7.66–7.62 (q, 1H), 7.60–7.55 (q, 1H), 7.21 (s, 1H), 6.81 (s, 1H), 5.00–4.98 (m, 1H), 4.82–4.71 (m, 3H), 3.90–3.49 (m, 16H), 3.30–3.25 (m, 4H), 2.94–2.71 (m, 4H), 2.25–1.65 (m, 20H). ESI-MS (Positive): calcd. for $C_{52}H_{57}N_9O_7$: 919.44, found: 942.6 ($[M + Na]^+$).

C343-Pro8-OtBu (reference compound)

Coumarin343 (285 mg, 1.0 mmol), H-Pro8-OtBu (936 mg, 1.1 mmol), and HATU (494 mg, 1.3 mmol) were dissolved in anhydrous DMF (30 mL), then DIPEA (1.0 mL) was added. This solution was stirred for 1 h at 0 °C and then 20 h at room temperature. The distilled water was added into the reaction mixture, and then the product was extracted by chloroform. This solution was dried over sodium sulfate and was evaporated to dryness under reduced pressure. The crude product was purified by silica-gel column chromatography using chloroform:methanol (19:1 v/v) as eluent. The product (C343-Pro8-OtBu) was obtained as yellow powder (950 mg, 0.85 mmol, 85%). ^{1}H NMR (400 MHz, $CDCl_3$) δ: 7.79 (s, 1H), 6.81 (s, 1H), 4.80–4.71 (m, 7H), 4.44–4.41 (m, 1H), 3.92–3.26 (m, 16H), 3.30–3.26 (m, 4H), 2.94–2.71 (m, 4H), 2.25–1.65 (m, 36H), 1.41 (s, 9H).

C343-Pro8-OH

C343-Pro8-OtBu (871 mg, 0.78 mmol) was dissolved in 4M HCl/dioxane solution (30 mL). This solution was kept for 15 h at room temperature and then evaporated to dryness under reduced pressure. The product (C343-Pro4-OH) was obtained as yellow powder (812 mg, 0.76 mmol, 98%). ^{1}H NMR (400 MHz, $CDCl_3$) δ: 7.81 (s, 1H), 6.84 (s, 1H), 4.81–4.59 (m, 7H), 4.41–4.32 (m, 1H), 3.75–3.47 (m, 16H), 3.30–3.26 (m, 4H), 2.95–2.73 (m, 4H), 2.37–1.82 (m, 36H).

C343-Pro8-phen

C343-Pro8-OH (637 mg, 0.60 mmol), 5-(piperazin-1-yl)-1,10-phenanthroline hydrochloride (phen, 300 mg, 1.0 mmol), HOBt (153 mg, 1.0 mmol), and HATU (380 mg, 1.0 mmol) were dissolved in anhydrous DMF (30 mL), then DIPEA (1 mL) was added. This solution was stirred for 1 h at 0 °C and then 20 h at room temperature. The distilled water was added into the reaction mixture, and then the product was extracted by chloroform. This solution was dried over sodium sulfate and was evaporated to dryness under reduced pressure. The crude product was purified by aluminum column chromatography using chloroform:methanol (98:2 v/v) as eluent. The product (C343-Pro8-phen) was obtained as yellow powder (185 mg, 0.14 mmol, 23%). ^{1}H NMR (400 MHz, $CDCl_3$) δ: 9.19–9.18 (d, 1H), 9.08–9.01 (d, 1H), 8.57–8.54 (d, 1H), 8.13–8.11 (d, 1H), 7.79 (s, 1H), 7.66–7.62 (q, 1H), 7.60–7.55 (q, 1H), 7.21 (s, 1H), 6.81 (s, 1H), 5.01–4.98 (m, 1H), 4.82–4.71 (m, 7H), 3.92–3.50 (m, 24H), 3.30–3.25 (m, 4H), 2.94–2.71 (m, 4H), 2.25–1.65 (m, 36H).

BTQphenBoc (reference compound)

2-(Benzo[*b*]thiophen-2-yl)quinoline (2.17 g, 8.30 mmol) and $IrCl_3 \cdot 3H_2O$ (1.44 g, 4.1 mmol) were dissolved in 2-ethoxyethanol (100 mL) and distilled water (30 mL) and then the solution was heated at reflux for 15 h. After cooling, the precipitate formed was filtered to give a chloro-briged dimer of BTQ washed thoroughly with methanol and *n*-hexane. *tert*-Butyl 4-(1,10-phenanthrolin-5-yl)piperazine-1-carboxylate (250 mg, 0.69 mmol) and chloro-bridged dimer of BTQ (450 mg, 0.3 mmol,) were dissolved in tetrahydrofuran (50 mL) and methanol (50 mL), then the solution was refluxed at 4 h. After cooling, KPF_6 (130 mg, 0.70 mmol) was added to the solution and stirred at 1h. The solution was evaporated to dryness under reduced pressure. The crude product was purified by aminopropyl-functionalized silica-gel column chromatography using chloroform:methanol (98:2 v/v) as eluent. The product (BTQphenBoc) was obtained as red powder. ^{1}H NMR (400 MHz, $CDCl_3$) δ: 8.65–8.63 (d, 2H), 8.45-8.43 (d, 2H), 8.15–8.11 (t, 2H), 7.95–7.83 (m, 5H), 7.78–7.74 (q, 1H), 7.57–7.53 (d, 2H), 7.32 (s, 1H), 7.17–7.10 (m, 4H), 6.91-6.80 (dd, 2H), 6.68–6.70 (m, 4H), 6.33–6.31 (d, 2H), 3.73–3.60 (m, 4H), 3.10–2.95 (m, 4H), 1.45 (s, 9H).

2. Spectral Properties of Reference Compounds

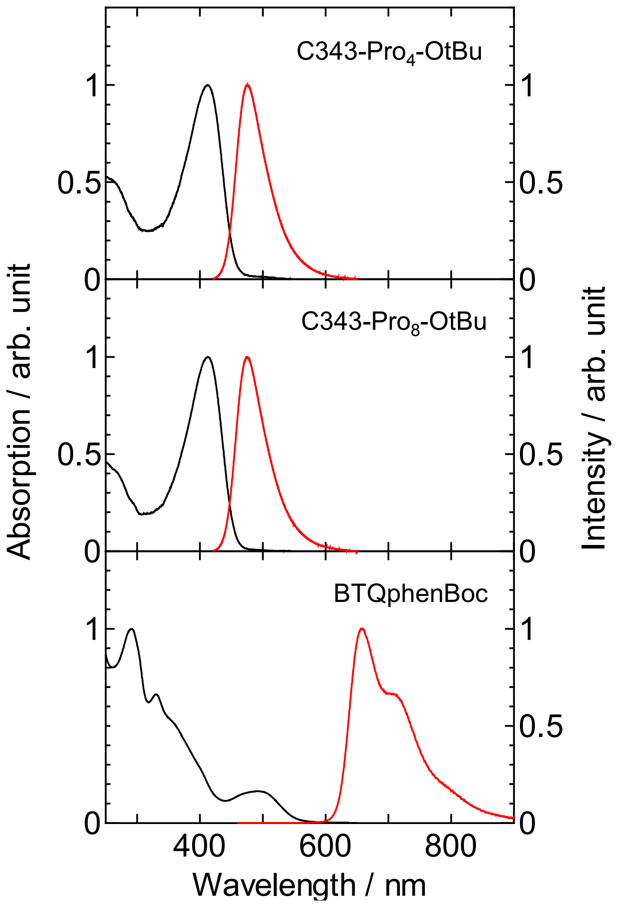

Figure S1. Absorption (black line) and emission (red line) spectra of C343-Pro4-OtBu, C343-Pro8-OtBu, and BTQphenBoc in MeCN.

3. Phosphorescence Lifetimes of RP2 in Living Cells

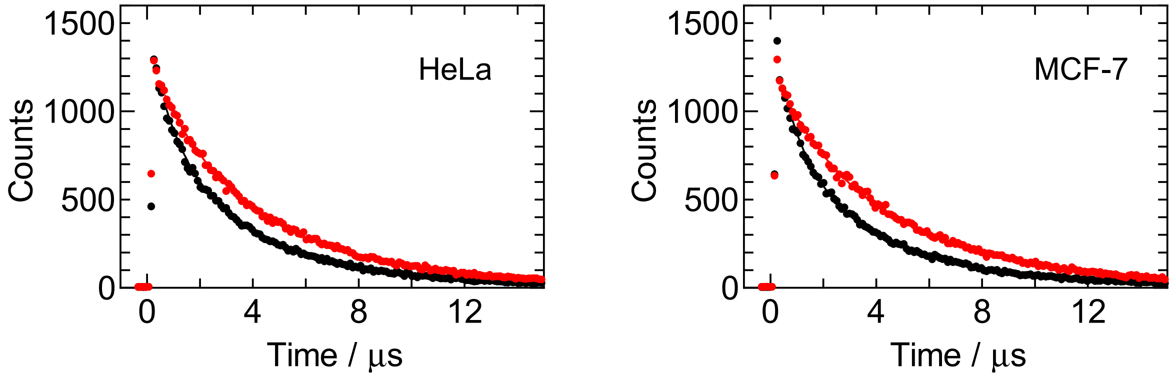

Figure S2. Phosphorescence decay curves of **RP2** in HeLa and MCF-7 cells under 21% (black) and 2.5% (red) O_2 conditions.

Table S1. The phosphorescence lifetimes (τ_p), the pre-exponential factors (A) and the intensity- averaged lifetimes ($<\tau_p>$) of **RP2** in HeLa and MCF-7 cells under 21% and 2.5% O_2 conditions.

Cells	O_2/%	τ_{p1}/μs	τ_{p2}/μs	A_1/%	A_2/%	$<\tau_p>$/μs
HeLa	21	1.04	3.93	39	61	2.80
	2.5	1.85	4.81	26	74	4.05
MCF-7	21	0.93	3.86	42	58	2.62
	2.5	1.98	4.98	21	79	4.34

© 2015 by the authors; licensee MDPI, Basel, Switzerland. This article is an open access article distributed under the terms and conditions of the Creative Commons Attribution license (http://creativecommons.org/licenses/by/4.0/).
